# Gas6 Attenuates Sepsis-Induced Tight Junction Injury and Vascular Endothelial Hyperpermeability *via* the Axl/NF-κB Signaling Pathway

**DOI:** 10.3389/fphar.2019.00662

**Published:** 2019-06-13

**Authors:** Jingjing Ni, Miaotong Lin, Yangjie Jin, Jiajia Li, Yayong Guo, Jindong Zhou, Guangliang Hong, Guangju Zhao, Zhongqiu Lu

**Affiliations:** Emergency Department, The First Affiliated Hospital of Wenzhou Medical University, Wenzhou, China

**Keywords:** Gas6, multi-organ dysfunction syndrome, vascular endothelial hyperpermeability, tight junctions, Axl/NF-κB

## Abstract

Vascular endothelial functional dysregulation and barrier disruption are involved the initiation and development of sepsis. Growth arrest-specific protein 6 (Gas6), one of the endogenous ligands of TAM receptors (Tyro3, Axl, and Mertk), is confirmed to have beneficial functions in hemostasis, inflammation, and cancer growth. Here, we demonstrated the protective effects of Gas6 on multi-organ dysfunction syndrome (MODS) in sepsis and the underlying mechanisms. We investigated Gas6-ameliorated MODS by inhibiting vascular endothelial hyperpermeability in a mouse model of sepsis. Additionally, *in vitro*, under lipopolysaccharide (LPS) stimulation in vascular endothelial cells, Gas6 attenuated vascular endothelial hyperpermeability by reinforcing the tight junction proteins occludin, zonula occludens-1 (ZO-1), and claudin5. Furthermore, Gas6 substantially suppressed NF-κB p65 activation. In addition, blocking the Gas6 receptor, Axl, partially reduced the protective effect of Gas6 on the vascular endothelial barrier and diminished the inhibitive effect of Gas6 on NF-κB p65 activation. Taken together, this study suggests that Gas6 has a protective effect on MODS in sepsis by inhibiting the vascular endothelial hyperpermeability and alteration of tight junction and that the Axl/NF-κB signaling pathway underlies these effects.

## Introduction

Sepsis is a life-threatening organ dysfunction caused by a dysregulated host response to infections, which is a major reason for intensive care unit (ICU) admission, resulting in 17% hospital mortality during the last decade in some high-income countries, and mortality in sepsis is most often attributed to multi-organ dysfunction syndrome (MODS) (Fleischmann et al., 2016; Singer et al., 2016; Hattori et al., 2017). Severe vascular endothelial barrier failure is associated with sepsis, leading to tissue edema and anasarca, leukocyte adhesion and transmigration, and hemostasis imbalance and vasomotor tone alteration (Shapiro et al., 2010; Paulus et al., 2011; Deutschman and Tracey, 2014). This hyperpermeability of the endothelial barrier is identified as the key factor in progression to MODS during sepsis (Ince et al., 2016).

Gas6, a 75-kDa vitamin K-dependent protein, binds receptor tyrosine kinases of the TAM (Tyro3, Axl, and Mertk) family. The Gas6–TAM interaction then activates downstream signaling, which produces biological effects such as cell survival, growth, migration, proliferation, inflammation, and cancer growth (van der Meer et al., 2014; Wu et al., 2017). TAM receptors can be found in many cell types. Tyro3 receptors are mainly expressed in the central nervous system, and Axl receptors are expressed in endothelial cells, platelets, vascular smooth muscle cells, and others. Mertk receptors are mostly expressed in monocytes and platelets (van der Meer et al., 2014). Previous studies demonstrated that Gas6 exerted protective effects in sepsis-induced acute kidney injury and acute lung injury in mice (Giangola et al., 2013; Chen et al., 2016). However, how Gas6 alleviates MODS remains unclear. In our study, we first reported that the protective role of Gas6 on sepsis-induced MODS was related to the vascular endothelial permeability.

The vascular endothelial permeability is mediated by paracellular and transcellular pathways. Transcellular pathways, or transcytosis, transports albumin and some macromolecules across the endothelium in normal circumstances (Minshall and Malik, 2006; Dejana et al., 2009; Komarova and Malik, 2010). The paracellular pathway is controlled by endothelial junctions, which contains three protein complexes, namely, adherens junctions (AJs), tight junctions (TJs), and gap junctions (GJs). All of them play essential roles in barrier function and cell–cell communication. Our previous study illustrated that the plasma levels of TJ-associated proteins were positively associated with organ dysfunction in septic patients (Zhao et al., 2016), which illustrated that TJs may serve as a therapy target for MODS in sepsis. TJs are formed by several adhesive proteins, including occludin, claudins, junctional adhesive proteins (JAMs), and intracellular proteins, among which the most important include the zonula occludens (ZO) family proteins. Claudins and occludin bind to cytosolic ZO-1, ZO-2, and ZO-3 proteins and assemble a zipper-like structure at the border of endothelial cells (Dejana et al., 2009; Komarova et al., 2017). There were reports indicated that lipopolysaccharide (LPS), a type of Gram-negative bacterial endotoxin, induced endothelial cell barrier failure through modulation of NF-κB activation both *in vivo* and *in vitro* (Liu and Malik, 2006; Xu et al., 2010; Recoquillon et al., 2015). It is believed that NF-κB activation is involved in endothelial hyperpermeability.

In brief, our study is performed to investigate that Gas6 ameliorates MODS by inhibiting vascular endothelial hyperpermeability through reinforcing TJ and attenuates permeability in vascular endothelial cells *via* Axl/NF-κB signaling pathways.

## Materials and Methods

### Materials

Rabbit polyclonal anti-ZO-1, mouse monoclonal anti-occludin, and mouse monoclonal anti-claudin5 were obtained from Thermo Fisher Scientific (Waltham, MA, USA). Rabbit monoclonal anti-β-actin, anti-phospho-NF-κB p65, and anti-NF-κB p65 were purchased from Cell Signaling Technology (Danvers, MA, USA). Goat polyclonal anti-Axl, anti-Mertk, and rat monoclonal anti-Tyro3 antibodies were obtained from R&D Systems (Minneapolis, MN, USA). Rabbit polyclonal anti-phospho-Axl, anti-phospho-Mertk, and anti-phospho-Tyro3 were purchased from Affinity (Cincinnati, OH, USA). Horseradish peroxidase (HRP)-conjugated and Alexa Fluor-conjugated secondary antibodies were purchased from Biosharp (Hefei, China). Axl small interfering RNA (siRNA) or nonspecific control siRNA with the recommended transfection reagents were all from RiboBio (Guangzhou, China). LPS (O55:B5) was purchased from Sigma-Aldrich (Oakville, Ontario, Canada). The In Vitro Vascular Permeability Assay kit was obtained from Merck Millipore (Etobicoke, Ontario, Canada). Recombinant Mouse Gas6 protein was from R&D Systems (Minneapolis, MN, USA). The chemiluminescence (ECL) Western blot detection kit was from Wenke Biotechnology (Zhejiang, China).

### Cell Culture

Mouse aortic endothelial cells (MAECs) were derived and cultured as previously described (Wang et al., 2016). Briefly, cells were cultured in endothelial cell growth medium (Heidelberg, Germany) supplemented with 1% penicillin/streptomycin, 10% fetal bovine serum (FBS), and endothelial cell growth supplement at 37°C with 5% CO_2_ and were used from passage 2–3 when grown to confluent monolayers.

Human umbilical vein endothelial cells (HUVECs) were purchased from the Shanghai Institute of Cell Biology. HUVECs were grown in endothelial cell growth medium (Heidelberg, Germany) supplemented with 1% penicillin/streptomycin, 10% FBS, and endothelial cell growth supplement. Cells were cultured in an atmosphere of 5% CO_2_ at 37°C and used at passages 5–6.

Endothelial cells (ECs) were serum-starved (1% serum) for 6 h before treatment and divided into four groups randomly: 1) control group: cells without any stimulation; 2) LPS group: cells were stimulated with LPS (50 μg/L, 100 μg/L, and 200 μg/L) for 12 h; 3) Gas6 group: cells were incubated with Gas6 (200 ng/mL); and 4) Gas6 + LPS group: cells were pretreated with Gas6 (100 ng/mL, 200 ng/mL, and 400 ng/mL) for 2 h and then treated with LPS (200 μg/L) for 12 h. In some experiments, cells were transfected with Axl siRNA (siAxl) or nonspecific control siRNA (siNC) for 36 h before being treated with Gas6 and LPS as described.

In all experiments *in vitro*, both MAECs and HUVECs were subjected to transwell permeability assay while MAECs were used in Western blotting and HUVECs were used in immunofluorescence.

### Transwell Permeability Assay

An *in vitro* Vascular Permeability Assay kit was used to measure the passage of fluorescein isothiocyanate (FITC)-dextran across the endothelial monolayer according to the manufacturer’s protocol. Briefly, cells were seeded at 2 × 10^4^ cells/insert on the upper chamber with collagen coating for 2–3 days to reach full confluence. ECs were incubated with Gas6 for 2 h before being stimulated with LPS for 12 h. Then, 150 μL of FITC-dextran in basal media (1:40) was added to the upper chamber and allowed to permeate through the monolayers. After 45 min, 100 μL of samples was removed from both the upper and lower chambers for fluorescence determination using a fluorescence microplate reader (SpectraMax M2, CA, USA) with an excitation wavelength of 485 nm and an emission wavelength of 535 nm. The permeability of the endothelial monolayer was evaluated by the permeability coefficient of dextran calculated as follows: Pd = [A]/*t* × 1/*A* × *V*/[L], where [A] is the FITC-dextran concentration in the bottom chamber and [L] is the FITC-dextran concentration in the upper chamber, *A* refers to the area of the membrane in cm^2^, *V* is the volume of the bottom chamber, and *t* indicates time in minutes.

### Mice and Experimental Groups

Male C57BL/6 mice (20–25 g) were obtained from Shanghai Slack Laboratory Animal Center [license: SCXK (HU) 2012-0002]. Mice were acclimatized in an environment with adequate temperature and humidity and had free access to water and chow for 7 days. All experimental protocols were performed according to the National Institutes of Health Guide for the Care and Use of Laboratory Animals, and all methods were approved by the ethics committee of the Laboratory Animal Ethics Committee of Wenzhou Medical University. Mice were randomly divided into the sham group, Cecal ligation and puncture (CLP) + vehicle group, and sham + Gas6 6 μg group.

### Animal Model of Sepsis and Drug Administration

Cecal ligation and puncture were performed as described previously (Wichterman et al., 1980; Nemeth et al., 2009). In short, after anesthetization by amobarbital sodium (50 mg/kg), we ligated the cecum at the distal two-thirds by silk 4-0 and punctured it with an 18-gauge needle. A small amount of fecal content was squeezed from holes, and the cecum was returned to the abdominal cavity. After we closed the abdomen in two layers, 1 ml of sterile saline was administered. In the sham group, we located the cecum but did not perform the ligation and puncture. In the treatment group, after CLP, 6 μg of Gas6 was administered *via* the tail vein immediately, and in the CLP group, animals received the same volume of normal saline.

### Evans Blue Dye Extravasation Assay

Evans blue dye extravasation assays (Xu et al., 2001) were performed 1 day after CLP. In brief, 0.2 mL of Evans blue dye (2%) was injected *via* the tail vein. After 2 h, the anesthetized mice were perfused with saline until no more blue dye came out of the right atrium. The lung and kidney were removed and weighed. Each tissue was then incubated in formamide at 72°C for 3 days and measured by a spectrophotometer at an excitation wavelength of 610 nm and an emission wavelength of 680 nm after the suspension was centrifuged at 10,000*g* for 25 min. The Evans blue extravasation was calculated as μg/g of tissue using a standard curve.

### Histological Staining

Lung and kidney tissues were fixed in 10% formalin and then embedded in paraffin for sectioning into 4-μm-thick sections. Tissues were stained with hematoxylin–eosin. The slides were reviewed in a blinded manner under an optic microscope (NIKON Eclipse Ci, Japan). The severity of lung injury was scored from 0 (normal) to 3 (severe), based on the degree of neutrophil infiltration, hemorrhage, interstitial edema, and alveolar congestion. The total score was calculated by adding up the individual scores of each category. The pathological changes in the kidney tissue were scored with a semi-quantitative scale to evaluate changes. Tubular damage was defined as tubular epithelial swelling, brush border losing, vacuolization, cellular necrosis, cast formation, and desquamation. The degree of kidney damage was determined according to the percentage of damaged tubules per all tubules by the following criteria: 0 (none), 1 (< 10%), 2 (10–25%), 3 (25–50%), 4 (50–75%), and 5 (>75%).

### Western Blotting

Cells were treated as described previously and harvested by cold Radioimmunoprecipitation Assay (RIPA) lysis buffer containing protease inhibitors and phosphatase inhibitors. After centrifugation at 12,000*g* for 20 min at 4°C, the protein concentration was assayed using the Bicinchoninic acid (BCA) assay. Equal amounts of protein (30 μg) were separated by 10–12% sodium dodecyl sulfate (SDS)-Polyacrylamide gel electrophoresis (PAGE) and transferred onto 0.45-μm polyvinylidene fluoride (PVDF) membranes (Millipore, CA, USA). After the membranes were blocked with 5% fat-free dry milk in Tris-buffered saline containing Tween-20 (TBST), they were incubated with anti-Tyro3 (1:1,000), anti-Axl (1:1,000), anti-claudin-5 (1:500), anti-occludin (1:1,000), anti-ZO-1 (1:500), anti-NF-κB p65, anti-phospho-NF-κB (1:1,000), phospho-Tyro3, anti-phospho-Axl, and anti-phospho-Mertk (1:500) at 4°C overnight. Membranes were washed three times and incubated with the HRP-conjugated secondary antibodies. Protein bands were visualized by using ECL reagents and performed with the ChemiDicTM XRS+ Imaging System (Hercules, CA, USA). Densitometry analysis of each band was quantified using Image Lab software and normalized against those of β-actin protein in each sample.

### Immunofluorescence

Cells were fixed in 4% paraformaldehyde for 15 min at room temperature and then washed three times with phosphate-buffered saline (PBS). After the cells were blocked with 1% bovine serum albumin (BSA) for 60 min at room temperature, they were incubated with antibodies against NF-κB (1:400), anti-ZO-1 (1:50), anti-claudin-5 (1:50), and occludin (1:100) at 4°C overnight. After the samples were washed three times, they were incubated with goat anti-rabbit Alexa Fluor 584-conjugated secondary antibody (1:400) for 2 h at room temperature. The cells were then counterstained with 4′6-diamidino2-phenylindole (DAPI) (Solarbio, Beijing, China) for 5 min and washed three times. Finally, the cell images were visualized using a Nikon A1Rsi confocal microscope (Nikon, Japan).

### siRNA Preparation and Transfection

According to the manufacturer’s recommendations, cells were grown to 50% confluency and then washed with PBS twice before transfection with siRNA. Cells were transfected with 10 nM mouse Axl siRNA (siAxl) or nonspecific control siRNA (siNC) using riboFECT^™^ buffer. Cells were transfected for 36 h, and the silencing efficiency was confirmed by Western blotting.

### Statistical Analysis

Data are expressed as the mean ± SD. All data analyses were carried out using GraphPad Prism 7 (GraphPad Software, La Jolla, CA, USA). Statistical significance between different groups was assessed by one-way ANOVA followed by Dunnett’s multiple comparison tests. Comparisons between two groups were made using Student’s *t* test. *P* < 0.05 was considered statistically significant.

## Results

### Gas6 Mitigates Histological Changes in Kidney and Lung Tissues

**Figure 1:**
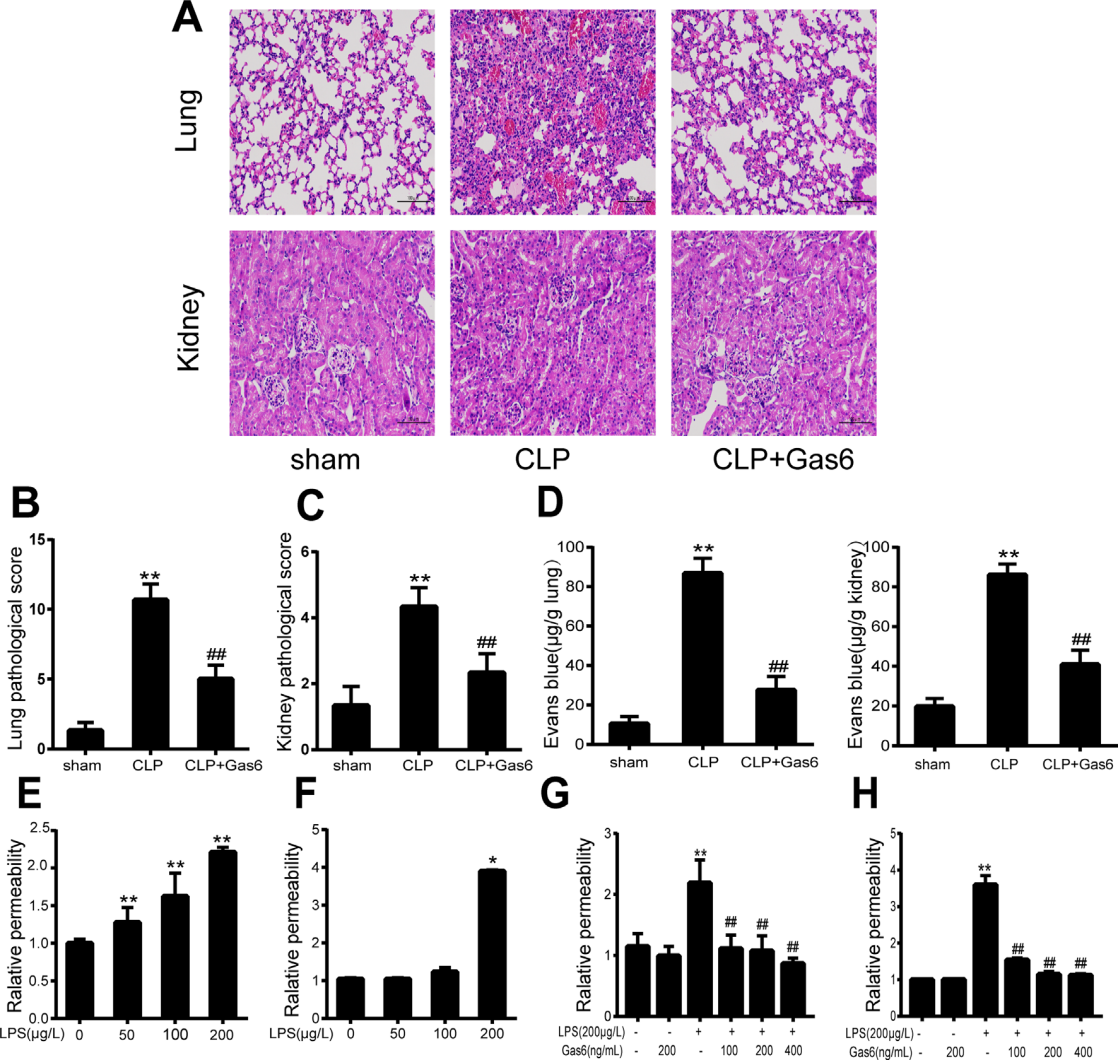
Gas6 ameliorated lung and kidney pathology in mice after CLP and prevented vascular hyperpermeability *in vitro* and *in vitro*. **(A)** Histological staining of lung and kidney tissue. Scale bar = 100 µm. Magnification was ×200. **(B, C)** Pathological scores of lung and kidney tissues of the Sham, CLP, and Gas6 treatment groups (*n* = 5, means ± SD, **P < 0.01 versus the Sham group, ^##^ *P* < 0.01 versus the CLP group). **(D)** The quantity of Evans Blue dye in the lung and kidney of the Sham, CLP, and Gas6 treatment groups (*n* = 6, means ± SD, **P < 0.01 versus the Sham group, ^##^ *P* < 0.01 versus the CLP group). **(E, F)** MAECs and HUVECs were treated with different concentrations of LPS (50 μg/L, 100 μg/L, and 200 μg/L) for 12 h. Dose-dependent effect of LPS on FITC-dextran permeability in MAECs and HUVECs (*n* = 3, means ± SD, **P < 0.01 versus the control group, *P < 0.05 versus the control group). **(G, H)** MAECs and HUVECs were treated with Gas6 (100 ng/mL, 200 ng/mL, and 400 ng/mL) for 2 h prior to treatment with LPS. Gas6 prevented LPS-induced vascular hyperpermeability in MAECs and HUVECs (*n* = 3, means ± SD, **P < 0.01 versus the control group, ^##^ *P* < 0.01 versus the LPS group).

To confirm the effect of Gas6 on the acute kidney and lung injury caused by CLP, histological staining was performed, and the representative histological microphotographs are shown in **Figure 1A**. Compared to the sham group, lung tissues showed marked pathological lesions characterized by edema, hemorrhage, and inflammatory infiltration in the CLP group, while treatment with Gas6 could mitigate the severity of pathological damages in a certain degree. Pathological scores were correspondingly elevated in the CLP group and significantly decreased in the Gas6-treated groups as quantified in **Figure 1B**. In kidney tissues, pathological lesions of tubular epithelial swelling, brush border injury, vacuolization, cellular necrosis, and cast formation were enhanced in the CLP group as compared with the sham group, whereas it was attenuated by Gas6 treatment. As quantified in **Figure 1C**, Gas6 treatment reduced the histological damage scores of the kidney.

### Gas6 Reduces Vascular Hyperpermeability *In Vivo*

To determine whether Gas6 ameliorated vascular hyperpermeability caused by sepsis, we performed an Evans blue dye extravasation assay. As shown in **Figure 1D**, 24 h after CLP, the mean Evans Blue dye accumulation in the lung and kidney was increased fourfold in the CLP group compared with the sham group. Remarkably, compared to the CLP group, the Gas6-treated group showed significantly reduced Evans blue dye accumulation. Gas6 alone did not affect Evans blue dye extravasation.

### Gas6 Attenuates LPS-Induced Vascular Endothelial Hyperpermeability *In Vitro*

The effects of LPS and Gas6 on endothelial permeability in MAECs and HUVECs were examined by analysis of Pd with FITC-dextran, an indicator of endothelial cell permeability. As shown in **Figure 1E**, LPS significantly increased the endothelial permeability in a dose-dependent manner within 12 h. Meanwhile, as shown in **Figure 1F**, LPS at a concentration of 200 μg/L significantly increased the HUVECs endothelial permeability within 12 h. In contrast, when MAECs and HUVECs were pretreated with Gas6 for 2 h and then challenged with LPS for another 12 h, Gas6 reduced permeability, and this reduction was substantial when Gas6 was administered at 400 ng/mL (**Figure 1G and H**).

### Gas6 Protects Tight Junction Injury Induced by LPS *In Vitro*

**Figure 2:**
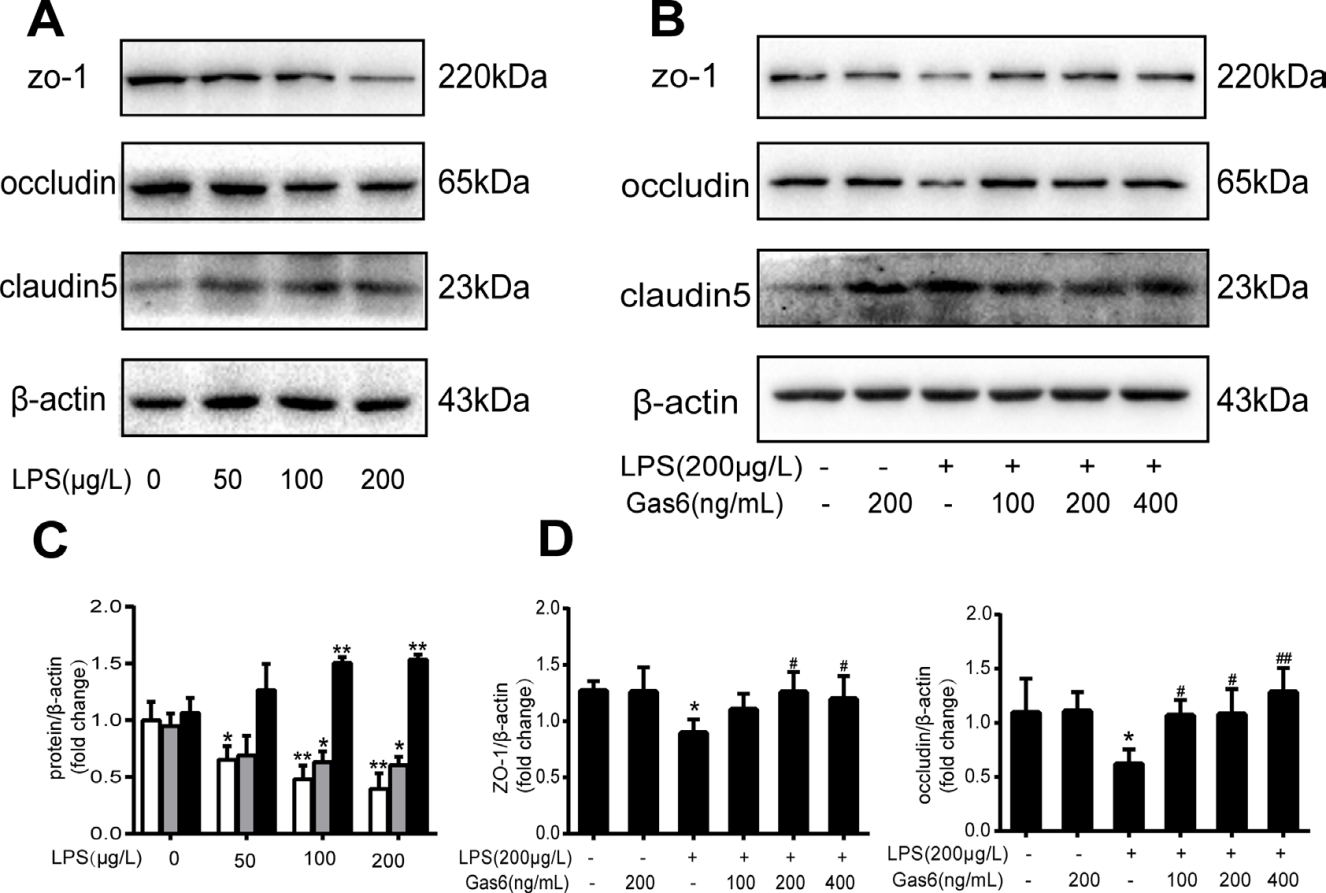
Gas6 prevented LPS-induced tight junction protein expression *in vitro*. MAECs were treated with different concentrations of LPS (50 μg/L, 100 μg/L, and 200 μg/L) for 12 h and treated with Gas6 (100 ng/mL, 200 ng/mL, and 400 ng/mL) for 2 h prior to treatment with LPS. **(A, B)** Representative image of Western blotting analysis of ZO-1, occludin, and claudin-5 in the control, LPS, and Gas6 treatment groups. β-Actin was used as the loading control and for band density normalization. **(C, D)** Quantification of Western blotting data for ZO-1, occludin, and claudin-5 protein (*n* = 3, means ± SD, **P < 0.01 versus the control group, *P < 0.05 versus the control group, ^##^ *P* < 0.01 versus the LPS group, ^#^ *P* < 0.05 versus the LPS group).

To confirm whether Gas6 alleviated the endothelial barrier by regulating TJ proteins, we examined the expression of ZO-1, occludin, and claudin5 by Western blotting. After treatment with different concentrations of LPS for 12 h, the expression levels of ZO-1 and occludin were markedly decreased in a dose-dependent manner. Occludin was significantly decreased when the LPS concentration was 200 µg/L, while the expression of ZO-1 was reduced at all concentrations. Interestingly, claudin5 was increased after treatment with LPS (**Figure 2A and C**).

**Figure 3:**
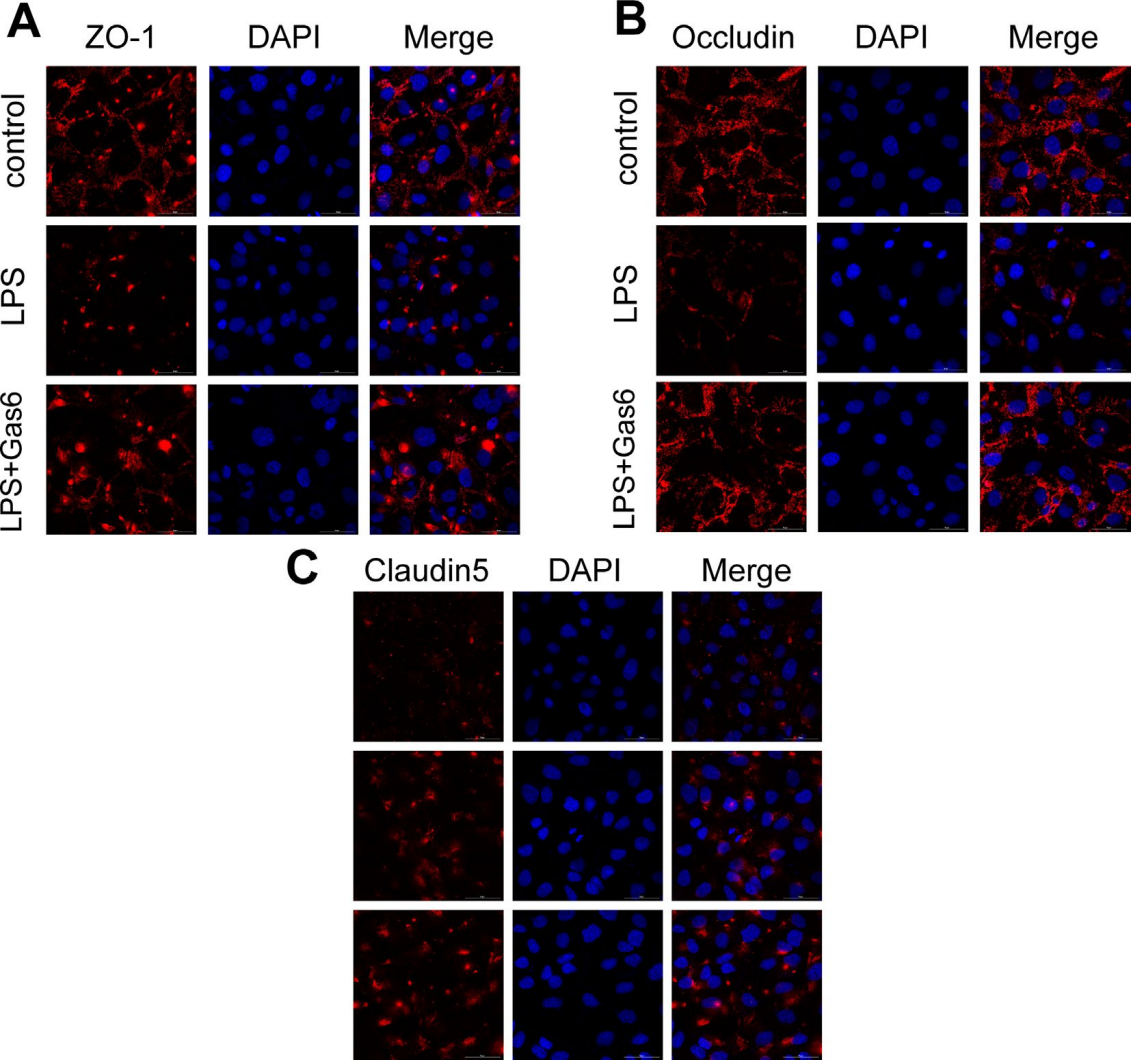
Gas6 prevented LPS-induced tight junction protein disassembly *in vitro*. HUVECs were pre-incubated with Gas6 (400 ng/mL) for 2 h prior to LPS (200 μg/L) exposure and then co-cultured for 12 h. Immunofluorescence staining of confluent HUVEC monolayers for ZO-1 **(A)**, occludin **(B)**, and claudin5 **(C)**. Scale bar = 50 μm. Magnification was ×600.

Based on the above results, LPS at a concentration of 200 μg/L was used for the following studies. Then, MAECs were incubated with different concentrations of Gas6 for 2 h before LPS stimulation. As shown in **Figure 2B and D**, we found that ZO-1 and occludin protein levels were completely improved at all concentrations of Gas6, and there was no difference among the three concentrations. Notably, the expression of claudin5 was also obviously increased after treatment with Gas6. Therefore, we used LPS at 200 μg/L and Gsa6 at 400 ng/mL in the following experiments. Additionally, Gas6 did not change the TJ protein expression level when incubated alone. To clarify further the mechanism of TJ injury, immunofluorescence shows that the location and distribution of ZO-1 and occludin in the cell–cell junction areas were disrupted under LPS (200 μg/L) stimulation compared with the control, while Gas6 (400 ng/mL) attenuated the disruption obviously. Meanwhile, claudin5 was restored as well after treatment with Gas6 (400 ng/mL) (**Figure 3A–C**).

### Gas6 Inhibits NF-κB Activation Induced by LPS *In Vitro*

**Figure 4:**
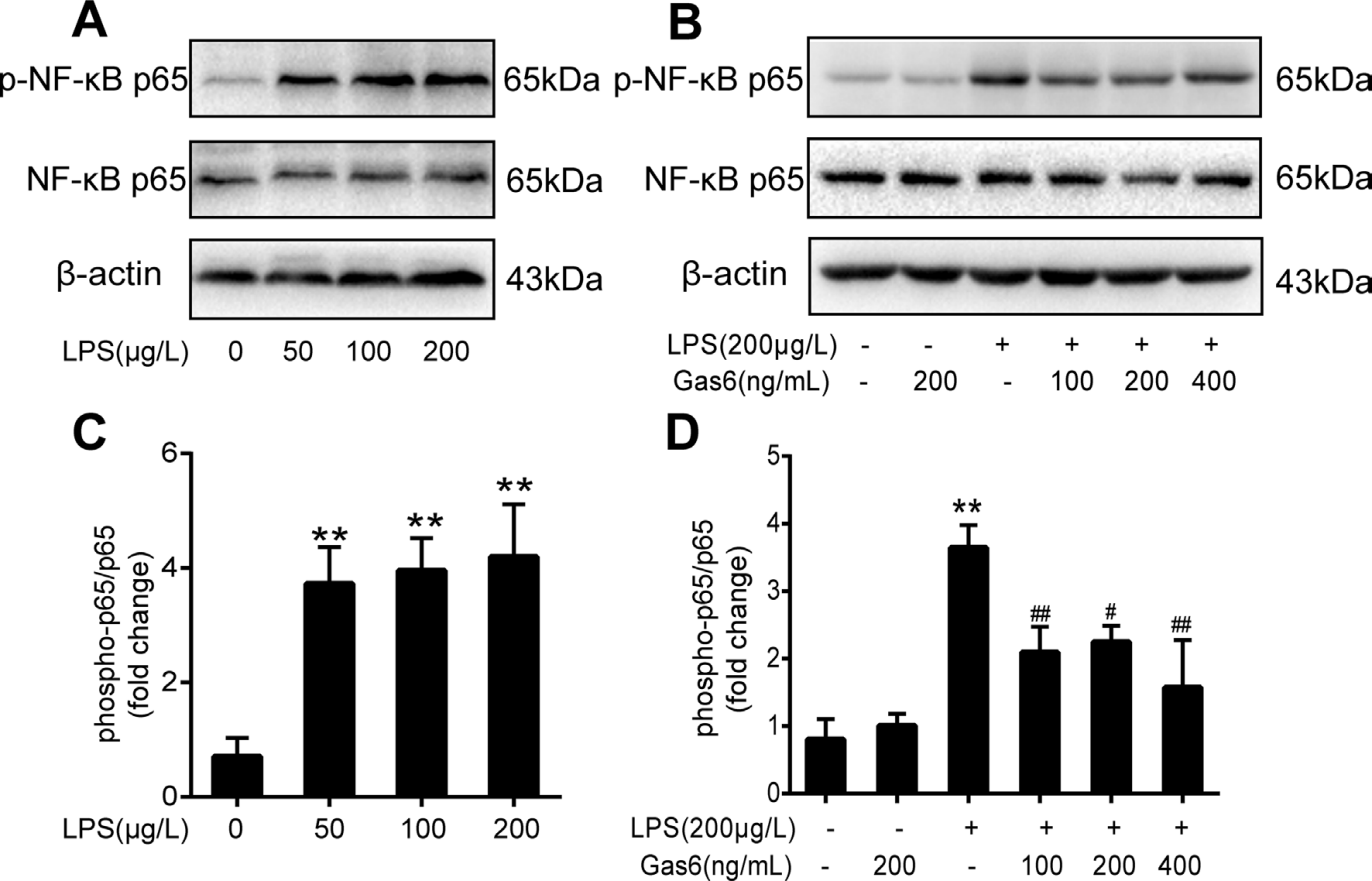
Gas6 inhibited the activation of NF-κB p65 under LPS stimulation. Cells were pretreated with Gas6 (100 ng/mL, 200 ng/mL, and 400 ng/mL) for 2 h prior to LPS exposure and co-cultured for 30 min. **(A, B)** The expression levels of phosphorylation of NF-κB p65 (p-NF-κB p65) and total NF-κB p65 were determined by Western blotting. **(C, D)** Quantification of Western blotting data for p-NF-κB p65 protein (*n* = 4, means ± SD, **P < 0.01 versus control group, ^##^ *P* < 0.01 versus the LPS group, ^#^ *P* < 0.05 versus the LPS group).

To determine the influence of Gas6 on NF-κB activation induced by LPS, we performed Western blotting of NF-κB. First, the phosphorylation of NF-κB p65 (p-NF-κB p65) and total NF-κB p65 was examined after different concentrations of LPS stimulation for 30 min. As shown in **Figure 4A and C**, LPS increased the phosphorylation of NF-κB p65 in a dose-dependent manner. As shown in **Figure 4B and D**, phosphorylation of NF-κB p65 was abrogated when cells were pretreated with Gas6 (100 ng/mL, 200 ng/mL, and 400 ng/mL) for 2 h prior to LPS exposure and co-cultured for 30 min. In addition, treatment with Gas6 alone did not affect the NF-κB activity.

### Gas6 Prevents LPS-Induced Endothelial Hyperpermeability Depending on the Axl/NF-κB Pathway

**Figure 5:**
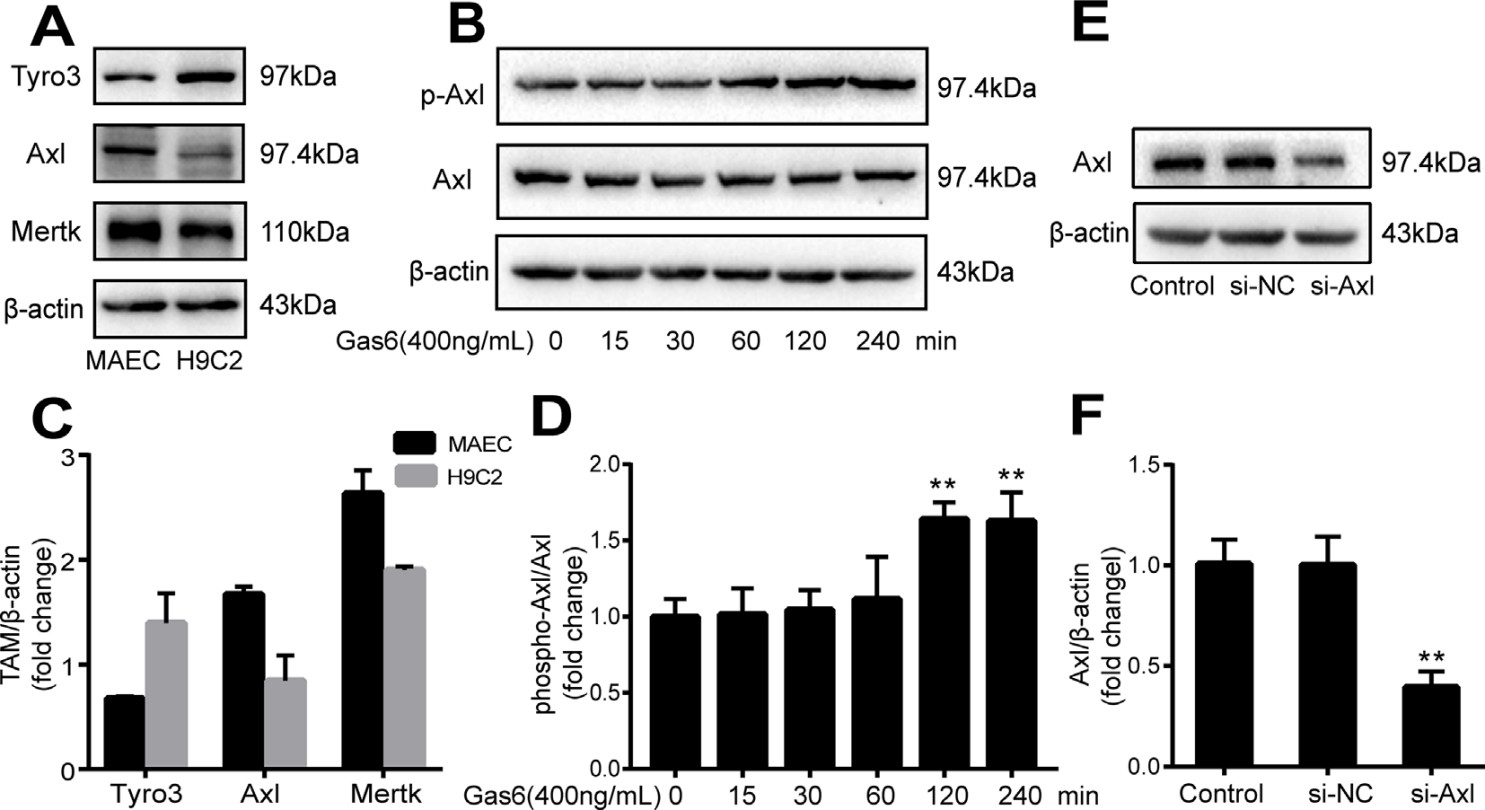
TAM receptor expression in MAECs and Axl siRNA transfection efficiency. **(A, C)** Western blotting analysis of the expression of TAM receptors (Tyro3, Axl, and Mertk) in MACEs (*n* = 3, means ± SD). **(B, D)** Axl was activated when MAECs were incubated with Gas6; Western blotting was used to examine the Axl phosphorylation expression (*n* = 3, means ± SD, **P < 0.01 versus the control group). **(E, F)** MAECs were transfected with Axl siRNA (siAxl) or nonspecific control siRNA (siNC) for 36 h; Western blotting was used to examine the protein expression of Axl after MAECs transfected with siAxl. β-Actin was used as the loading control and for band density normalization (*n* = 3, mean ± SD, **P < 0.01 versus the siNC group).

**Figure 6:**
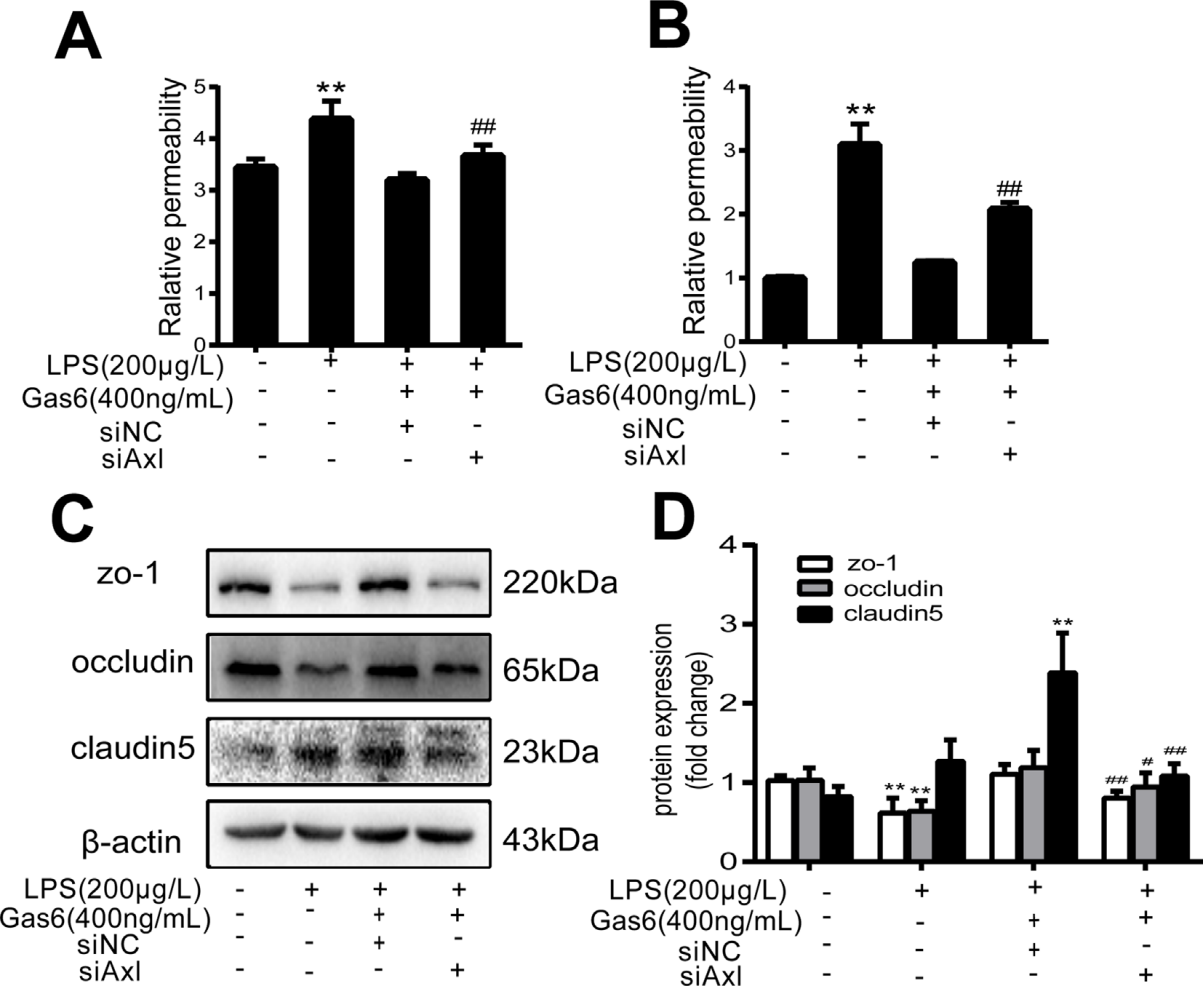
Blockage of Axl diminished the protective effect of Gas6 on LPS-induced vascular hyperpermeability. **(A, B)** MAECs and HUVECs were transfected with siAxl and then pre-incubated with Gas6 (400 ng/mL) for 2 h prior to LPS (200 μg/L) exposure and then co-cultured for 12 h; effect of Gas6 on FITC-dextran permeability in MAECs and HUVECs was assessed (*n* = 3, means ± SD, **P < 0.01 versus control group, ^##^ *P* < 0.01 versus the siNC group). **(C)** MAECs were transfected with siAxl and then pre-incubated with Gas6 (400 ng/mL) for 2 h prior to LPS (200 μg/L) exposure and then co-cultured for 12 h. Western blot was used to examine the protein expression of ZO-1, claudin 5, and occludin. β-Actin was used as the loading control and for band density normalization. **(D)** Quantification of Western blotting data for ZO-1, occludin, and claudin-5 protein (*n* = 4, means ± SD, **P < 0.01 versus control group, *P < 0.05 versus the control group, ^##^ *P* < 0.01 versus the siNC group, ^#^ *P* < 0.05 versus the siNC group).

**Figure 7:**
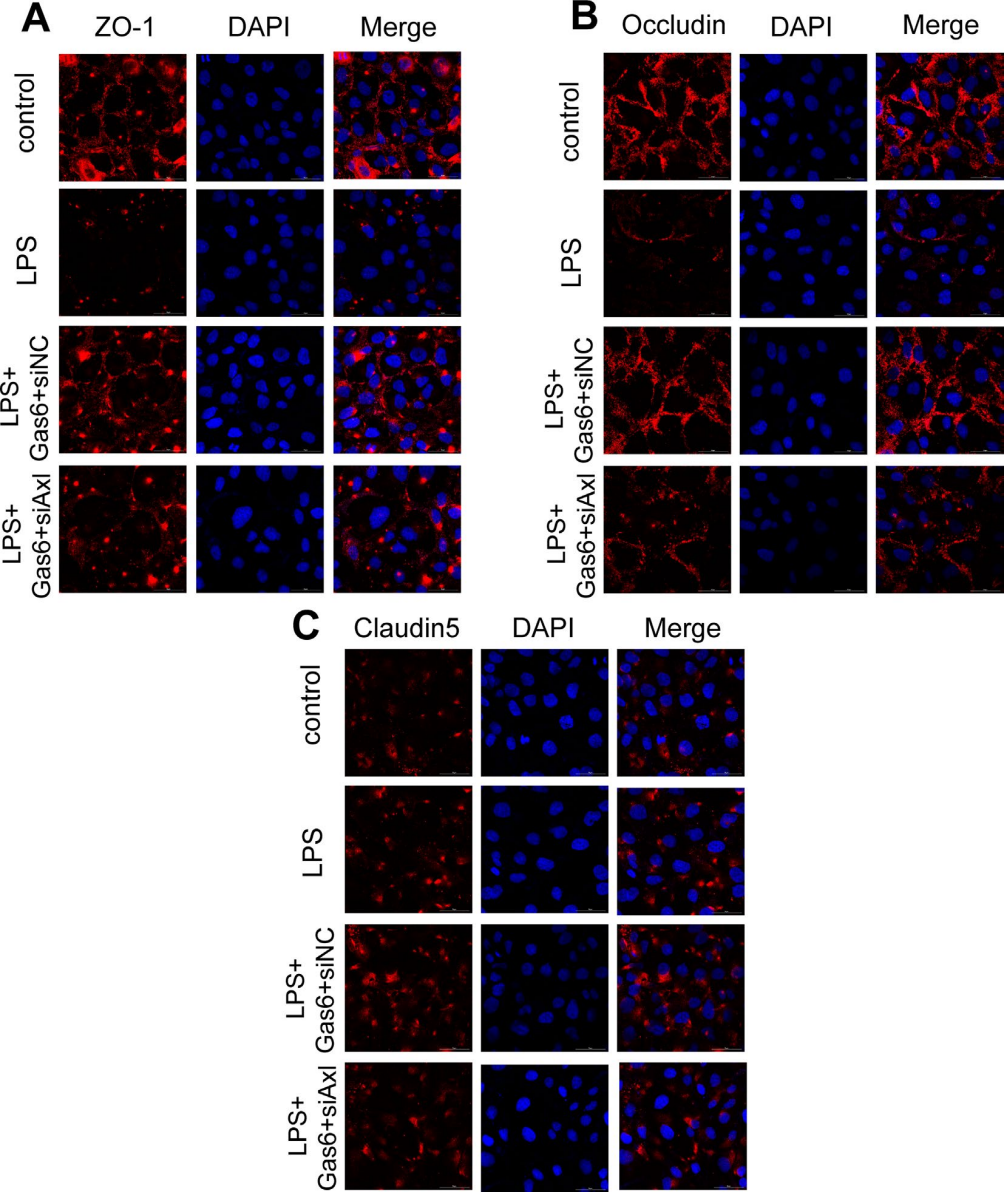
Blockage of Axl diminished the protective effect of Gas6 on LPS-induced tight junction injury. **(A–C)** Immunofluorescence staining of ZO-1 **(A)**, occluding **(B)**, and claudin-5 **(C)** was examined by a confocal microscope after HUVECs were transfected with siAxl and then pre-incubated with Gas6 (400 ng/mL) for 2 h prior to LPS (200 μg/L) exposure and then co-cultured for 12 h. Scale bar = 50 µm. Magnification was ×600.

**Figure 8:**
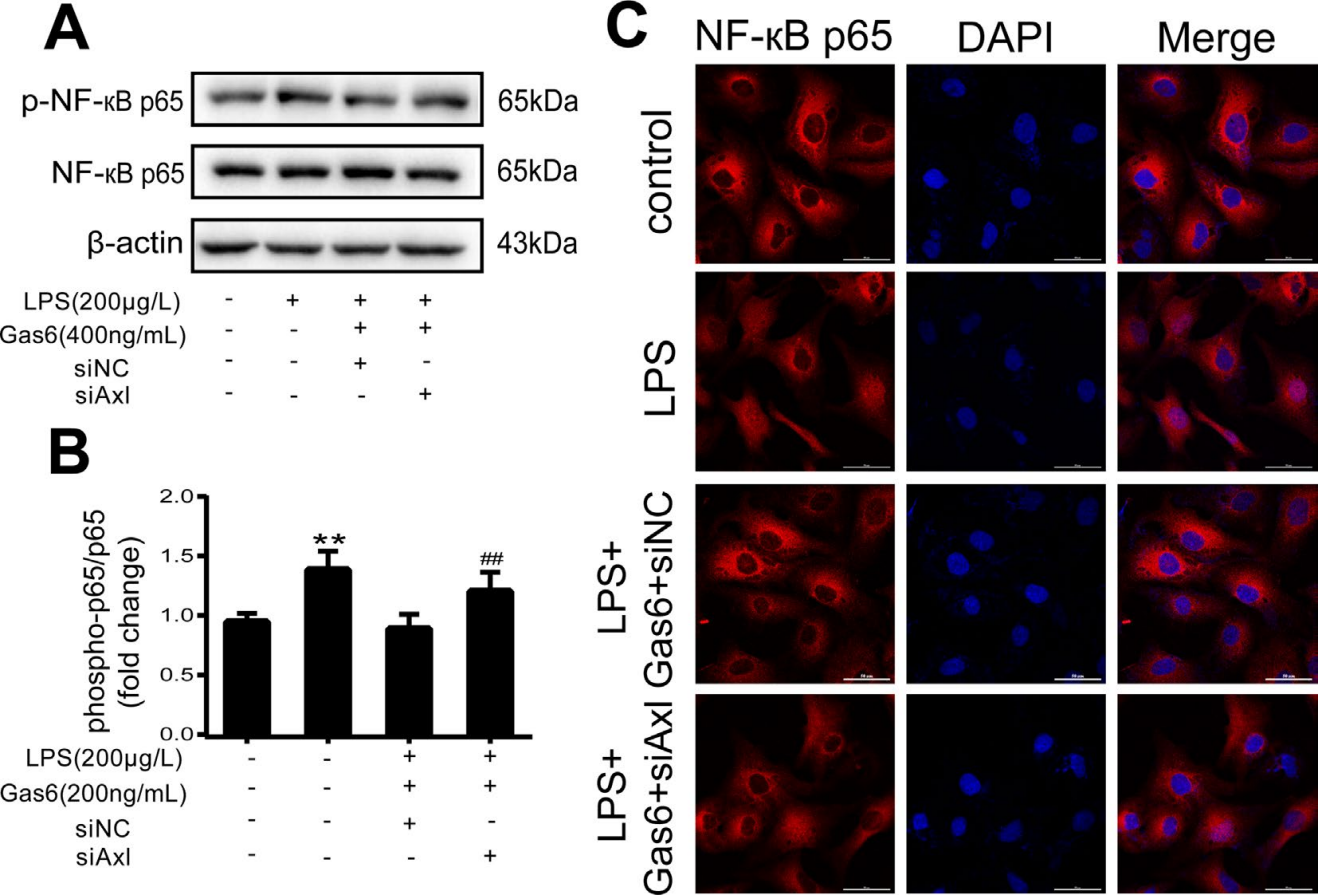
Blockage of Axl diminished the inhibitive effect of Gas6 on NF-κB p65 activation. **(A)** MAECs were transfected with siAxl and then pre-incubated with Gas6 (400 ng/mL) for 2 h prior to LPS (200 μg/L) exposure and then co-cultured for 30 min. **(B)** Western blotting was used to examine the protein expression of p-NF-κB p65 and NF-κB p65. NF-κB p65 was used as the loading control and for band density normalization (*n* = 3, means ± SD, **P < 0.01 versus control group, ##*P* < 0.01 versus the siNC group). **(C)** Immunofluorescence staining of NF-κB p65 was examined by a confocal microscope after HUVECs were transfected with siAxl and then pre-incubated with Gas6 (400 ng/mL) for 2 h prior to LPS (200 μg/L) exposure and then co-cultured for 30 min. Scale bar = 50 µm. Magnification was ×600.

The expression of TAM receptors (Tyro3, Axl, and Mertk) in MAECs was determined by Western blotting. Cardiac muscle cell H9C2 was used as a positive control. We found that MAECs expressed all three receptors simultaneously (**Figure 5A and C**). However, only Axl was activated when MAECs were incubated with Gas6. We detected obvious Axl phosphorylation within 15 min (**Figure 5B and D**). There was no significant change in the other two receptors (data not shown). To further confirm whether Gas6 attenuated LPS-induced vascular endothelial hyperpermeability *via* Axl/NF-κB signaling pathways *in vitro*, we transfected endothelial cells with siAxl or siNC molecules. The transfection efficiency was examined by Western blotting. Compared with cells transfected with siNC, cells transfected with siAxl showed a 39.5% reduction in Axl expression (**Figure 5E and F**). The reduction in Axl expression significantly inhibited the protective effect of Gas6 in permeability (**Figure 6A and B**) when compared to that of the siNC group. Real-time PCR was also used to examine the Axl siRNA transfection efficiency after MAECs transfected with siAxl (**Supplementary Figure 1**). Furthermore, Gas6 significantly increased ZO-1, occludin, and claudin5 expression, which was inhibited when Axl expression was downregulated using siAxl compared with that of the siNC group **(Figure 6C and D**). As shown in **Figure 7A and B**, the breakdown of ZO-1 and occludin was markedly restored after Gas6 (400 ng/mL) treatment compared to the LPS group (200 μg/L), and the protection of ZO-1 and occludin was decreased when transfected with siAxl. Meanwhile, the fluorescence density of claudin5 was increased after treatment with Gas6, while it was decreased when transfected with siAxl (**Figure 7C**). In addition, knocking down Axl markedly reduced Gas6’s inhibitive effect on phosphorylation levels of NF-κB stimulated by LPS (**Figure 8A and B**). To further confirm the NF-κB activation induced by LPS, we detected NF-κB p65 translocation in HUVECs by immunofluorescence. As shown in **Figure 8C**, LPS obviously induced the nuclear localization of NF-κB p65, while Gas6 strongly mitigated the NF-κB p65 nuclear transfer induced by LPS, and the effect was attenuated with siAxl transfected.

## Discussion

Sepsis has been defined as a systemic inflammatory syndrome, and the immunoinflammatory system is believed to be responsible for the pathogenesis of MODS. However, therapeutic strategies aimed at alleviating the inflammatory response have only shown modest clinical benefits (Hattori et al., 2017).

Gas6 is a vitamin K-dependent protein that is an interesting target in biomedicine due to its role in inflammation, hemostasis, and cancer (Bellido-Martín and de Frutos, 2008). In fact, many studies have investigated the effect of Gas6 on sepsis. Gas6 was present at high levels in plasma during sepsis and correlated well with organ dysfunction (Borgel et al., 2006; Gibot et al., 2007; Ekman et al., 2010; Yeh et al., 2017). In line with our findings (**Figure 1**), some studies have shown that Gas6 can effectively reduce organ injury in sepsis (Giangola et al., 2013; Chen et al., 2016).

Vascular endothelial cells separate circulating fluid and inflammatory cells from tissues. In sepsis, activation cascades are initiated by the release of endotoxin or proinflammatory cytokines in endothelial cells, which results in vascular endothelial barrier failure and increased vascular permeability (Paulus et al., 2011; Ince et al., 2016). More seriously, the dysregulation of the vascular endothelial barrier can then lead to microcirculation impairment and MODS (Fisher and Linder, 2017). Growing evidence has shown that endothelial barrier activation and disruption play a principal role in the pathophysiology of sepsis and have been suggested to be a predictor of mortality in sepsis (Huet et al., 2011;Julie et al., 2013). In this study, we found that Gas6 decreased vascular hyperpermeability induced by CLP *in vivo* and LPS *in vitro* (**Figure 1**). Therefore, we hypothesized that Gas6 reduced organ injury by protecting vascular endothelial barrier and vascular permeability.

Vascular permeability involves two distinct pathways: transcellular and paracellular routes. Two routes maintain endothelial permeability and tissue fluid homeostasis by regulating the exchange of plasma proteins, solutes, and water. The transcellular route is mostly mediated by caveolae-mediated vesicular transport, which predominantly occurs from the endothelial luminal surface to the basal surface (Komarova and Malik, 2010). On the other hand, the paracellular route is controlled by the endothelial junctions (Bazzoni, 2005; Dejana et al., 2009). AJs, TJs, and GJs all play important roles in vascular integrity as well as in vascular permeability (Wallez and Huber, 2008). In fact, GJs are intercellular channels that are responsible for direct electric and chemical communication between endothelial cells. AJs are localized more basally and control vessel maturation and stability as well as limiting paracellular permeability. Finally, the major function of TJs in the endothelium is to restrict paracellular permeability, and they occupy the most apical position. TJs consist of integral membrane proteins and intracellular proteins. Integral membrane proteins, such as occludin, claudins, and JAM-A, combine with the intracellular protein ZO-1. In turn, ZO-1 binds F-actin to change the paracellular permeability (Bazzoni, 2005; Dejana et al., 2009; Komarova et al., 2017). Yu, H. et al. found that interleukin-8 (IL-8) increased endothelial permeability by downregulating TJ protein expression and rearrangement of the distribution of TJs (Yu et al., 2013). Capaldo, C.T. et al. also demonstrated that the endothelial permeability increased and TJ proteins became disorganized and discontinuous after proinflammatory cytokine TNF-α and IFN-γ treatment (Capaldo and Nusrat, 2009). In this study, we showed that pretreatment with Gas6 up-regulated occludin and ZO-1 protein levels after LPS treatment in MAECs, while the protein claudin-5 was increased both in LPS stimulation and Gas6 treatment. It is not clear why this difference occurred, and it may be related to differences in the cell line or primary culture and the cell culture conditions (**Figure 2**). Immunofluorescence shows that the breakdown of ZO-1, occludin, and claudin5 were markedly restored after Gas6 (400 ng/mL) treatment (**Figure 3**). Together, Gas6 was shown to decrease vascular endothelial permeability by up-regulating and rearrangement of TJs through the paracellular pathway.

TAM receptors can be found in a variety of cell types and tissues (Shafit-Zagardo et al., 2018). Gas6 has different affinities with three receptors: Axl, Tyro3, and Mer (Axl ≥ Tyro3 ≥ Mer) (van der Meer et al., 2014). As receptor tyrosine kinases, TAM receptors are activated when Gas6 binds, followed by homodimerization, tyrosine autophosphorylation, and phosphorylation of downstream signaling pathways, such as the phosphoinositide 3-kinase (PI3K), extracellular regulated protein kinases (ERK), and NF-κB pathways. Cells express diverse TAM receptors, which have various downstream signaling pathways and different cell biological activities (Kimani et al., 2016). In the present study, we showed that all three TAM receptors were expressed in the MAECs, but only Axl was activated following Gas6 treatment (**Figure 5**).

Various signals, including G protein, protein kinase, and phosphatase, regulate TJ and vascular endothelial permeability. TNF-α increases bovine retinal endothelial cell (BREC) permeability through the protein kinase C (PKC) pathway in diabetic retinopathy (Aveleira et al., 2010). Similarly, IFN-γ and ILs can also lead to endothelial hyperpermeability, which is related to mitogen-activated protein kinase (MAPK) activation (Capaldo and Nusrat, 2009). Endothelial permeability is significantly increased in HUVECs after LPS treatment (Wang et al., 2017). Interestingly, all of these pathological processes can lead to the activation of NF-κB; that is to say, NF-κB activation is involved in endothelial barrier breakdown and endothelial hyperpermeability. Consistent with these results, our data demonstrated that LPS significantly activated NF-κB and Gas6 substantially suppressed NF-κB p65 activation (**Figure 4**).

To further confirm that Gas6 protects LPS-induced endothelial barrier disruption through the Axl/NF-κB signaling pathway *in vitro*, we transfected MACEs and HUVECs with siAxl or siNC. We found that TJ injury protected by Gas6 was attenuated following transfection with siAxl, and the effect of Gas6 on inhibition of NF-κB activation was decreased (**Figures 6**–**8**). Thus, we demonstrated Gas6-protected LPS-induced endothelial hyperpermeability *via* the Axl/NF-κB signaling pathway.

In conclusion, our results demonstrated that Gas6 ameliorated sepsis-induced MODS. Furthermore, the promising protective effect of Gas6 is mediated vascular endothelial hyperpermeability through reinforcing TJ *via* the Axl/NF-κB pathway. Therefore, Gas6 may be an interesting therapeutic strategy for recovery from sepsis and a suitable therapeutic option for sepsis.

## Data Availability Statement

The raw data supporting the conclusions of this manuscript will be made available by the authors, without undue reservation, to any qualified researcher.

## Ethics Statement

All experimental protocols were performed according to the National Institutes of Health Guide for the Care and Use of Laboratory Animals, and all methods were approved by the ethics committee of the Laboratory Animal Ethics Committee of Wenzhou Medical University.

## Author Contributions

ZL, GZ, and GH contributed to the conception and design of the manuscript. JN wrote the manuscript and conducted the entire study. ML, YG, and YJ assisted in the experiments performed in animals, H&E method, and semiquantitative analysis. JL and JZ coordinated the study. All authors agreed to be accountable for all aspects of the work.

## Funding

This work was supported by the Medical Health Science and Technology Major Project of the Zhejiang Provincial Health Commission (WKJ-ZJ-1724) and the National Natural Science Foundation (nos. 81571937 and 81772112).

## Conflict of Interest Statement

The authors declare that the research was conducted in the absence of any commercial or financial relationships that could be construed as a potential conflict of interest.

## Abbreviations

LPS, lipopolysaccharides; MODS, multi-organ dysfunction syndrome; Gas6, growth arrest-specific protein 6; TJs, tight junctions; TAM receptors, Tyro3, Axl, and Mertk; ZO-1, zonula occludens-1
