# Supplementary material for: Gas6 Attenuates Sepsis-Induced Tight Junction Injury and Vascular Endothelial Hyperpermeability via the Axl/NF-κB Signaling Pathway
Source: Front Pharmacol. 2019 Jun 13;10:662. doi: 10.3389/fphar.2019.00662 (PMC6585310; doi:10.3389/fphar.2019.00662)
Supplement: Supplementary file 1 [file DataSheet_1.pdf]

Supplementary Figures

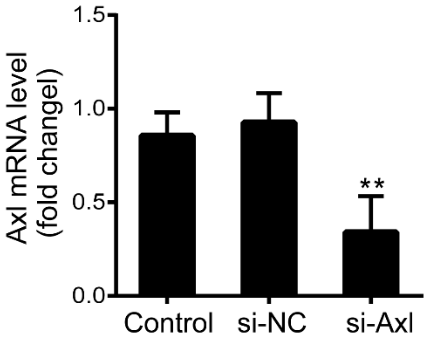

Supplementary Figure 1. Axl siRNA transfection efficiency. MAECs were transfected with Axl siRNA (siAxl) or nonspecific control siRNA (siNC) for 36 h, real-time PCR was used to examine the mRNA expression of Axl after MAECs transfected with siAxl. (n=3, mean ± SD, **P<0.01 versus the siNC group.)
